# Clinical Features and Outcome of Infective Endocarditis in a University Hospital in Romania

**DOI:** 10.3390/medicina57020158

**Published:** 2021-02-10

**Authors:** Emilia Elena Babeș, Diana Anca Lucuța, Codruța Diana Petcheși, Andreea Atena Zaha, Cristian Ilyes, Alexandru Daniel Jurca, Cosmin Mihai Vesa, Dana Carmen Zaha, Vlad Victor Babeș

**Affiliations:** 1 Department of Medical Disciplines, Faculty of Medicine and Pharmacy, University of Oradea, 410073 Oradea, Romania; babes.emilia@gmail.com (E.E.B.); babesvlad@gmail.com (V.V.B.); 2 Department of Cardiology, Clinical County Emergency Hospital of Oradea, 410169 Oradea, Romania; lucuta_diana@yahoo.com (D.A.L.); ilyescristian@yahoo.com (C.I.); 3 Department of Preclinical Disciplines, Faculty of Medicine and Pharmacy, University of Oradea, 410073 Oradea, Romania; petchesi_diana@yahoo.com (C.D.P.); alexjurca@yahoo.co.uk (A.D.J.); v_cosmin_15@yahoo.com (C.M.V.); danaczaha@gmail.com (D.C.Z.); 4 Faculty of Medicine, “Iuliu Hațieganu” University of Medicine and Pharmacy, 400000 Cluj Napoca, Romania

**Keywords:** endocarditis, etiology, correlations, complications, mortality

## Abstract

*Background and Objectives*: Characterization of patients with endocarditis regarding demographic, clinical, biological and imagistic data, blood culture results and possible correlation between different etiologic factors and host status characteristics. *Material and methods*: This is a retrospective observational descriptive study conducted on patients older than 18 years admitted in the past 10 years, in the Cardiology Clinic of the Clinical County Emergency Hospital Oradea Romania, with clinical suspicion of bacterial endocarditis. Demographic data, clinical, paraclinical investigations and outcome were registered and analyzed. *Results*: 92 patients with definite infective endocarditis (IE) according to modified Duke criteria were included. The mean age of patients was 63.80 ± 13.45 years. A percent of 32.6% had health care associated invasive procedure performed in the 6 months before diagnosis of endocarditis. Charlson’s comorbidity index number was 3.53 ± 2.029. Most common clinical symptoms and signs were: shortness of breath, cardiac murmur, fever. Sixty-six patients had native valve endocarditis, 26 patients had prosthetic valve endocarditis and one patient was with congenital heart disease. Blood cultures were positive in 61 patients. Among positive culture patient’s staphylococcus group was the most frequently involved: *Staphylococcus aureus* (19.6%) and coagulase negative *Staphylococcus* (18.5%). Most frequent complications were heart failure, acute renal failure and embolic events. *Conclusions: Staphylococcus aureus* IE was associated with the presence of large vegetations, prosthetic valve endocarditis and intracardiac abscess. Coagulase negative *Staphylococcus* (CoNS) infection was associated with prosthetic valve dysfunction. *Streptococcus gallolyticus* etiology correlated with ischemic embolic stroke and the presence of large vegetations. Cardiovascular surgery was recommended in 67.4% of patients but was performed only on half of them. In hospital death occurred in 33.7% of patients and independent predictors of mortality were congestive heart failure and septic shock.

## 1. Introduction

Despite the advances in diagnostic, surgical and critical care treatment endocarditis remains a life-threatening disease with an estimated in-patient mortality between 15–30% [1]. In recent years, the epidemiology of endocarditis has changed both with regards to pathogen and host as well as the bacteria’s spectrum. While in the past *Streptococcus viridans* used to be the most frequently encountered germ, *Staphylococcus aureus* has been gaining ground lately.

Patient demographic characteristics have also changed. Rheumatic valve disease and congenital heart disease used to be the main risk factors in the past; however, rates have been declining, degenerative valve disease currently being the main underlying disease. Therefore, patients are generally older and with multiple comorbidities [2]. The advances in cardiology and cardiac surgery with increased use of surgical and transcatheter prosthetic valve replacement, of implantable electronic devices, the increased use of venous catheters, contribute to the change in risk factor profile of the patients [3]. Increasing invasive therapeutic or/and diagnostic procedures in hospital, replacement of degenerative valve is responsible of bacteremia and finally healthcare-associated IE (infective endocarditis) representing about 30% of all endocarditis cases [1,2,4]. Healthcare-associated infective endocarditis is defined as either IE with onset of symptoms ≥48 h after admission to hospital or IE acquired after discharge from the hospital in association with a significant invasive procedure performed in the six months before diagnosis in at least two circumstances: during hospitalization and/or manipulation in a hospital setting (nosocomial healthcare-associated IE) and/or in patients with extensive out-of-hospital contact with healthcare interventions (non-nosocomial healthcare-associated IE).

Rapid identification of the etiological agent is important for successful management of the patient. Positive blood culture is a major diagnostic criterion for infective endocarditis but identification of etiology can fail due to several factors: prior administration of antibiotics before culture, fastidious or hard to culture infectious organisms such as HACEK or fungi, or poor microbiologic technique upon collection and cultures. Evaluating the spectrum of pathogens causing bacterial endocarditis in the community and the prevalence of pathogens in relation with different categories of patients can help in the initial management of patients which is empirical until identification of the etiological agent or in patients with culture negative endocarditis.

The aim of this study is characterization of patients with endocarditis regarding demographic, clinical and imagistic data and possible correlation between different etiologic factors and host status characteristics.

## 2. Materials and Methods

This is a retrospective observational descriptive study conducted on all patients older than 18 years with clinical suspicion of bacterial endocarditis admitted in the past 10 years, from January 2010 until December 2019, in the Cardiology clinic of the Clinical County Emergency Hospital Oradea Romania, a center without cardiovascular surgical facilities. Definite diagnosis of endocarditis was made if two major criteria, one major and three minor criteria or five minor modified Duke criteria were present [5].

Demographic data, patient history, clinical symptoms and signs, associated disease, antibiotic therapy, health care associated procedures six months prior admission and the department of initial admission were recorded. Charlson’s comorbidity index was calculated using a validated model [6]. Laboratory investigations such as CBC (complete blood count), erythrocyte sedimentation rate and C reactive protein, creatinine levels, results of blood cultures and serological tests were registered. Transthoracic echocardiography and in some patients also transesophageal echocardiography were performed.

Complications during hospitalization, evolution of patients, medical treatment, indication for surgery in emergency or planned and in hospital mortality were noted and analyzed.

All subjects gave their informed consent for inclusion before they participated in the study. The study was conducted in accordance with the Declaration of Helsinki, and the protocol was approved by the Ethics Committee of Clinical County Emergency Hospital Oradea, Bihor County (decision no 911/14.01.2020). All patients included in the study have signed the informed consent form at admission.

Statistical analysis was made using SPSS statistical package version 25. Categorical data was expressed as frequencies and percentages. Continuous data was expressed as mean ± SD. Relationship between quantitative variables was analyzed using Pearson bivariate correlation test and between categorical variable Spearman bivariate correlation test.

Intergroup comparison was made using Kruskal Wallis test for categorical data and one-way Anova for continuous data with post hoc analysis to determine which of these groups differ from each other. Binary simple logistic regression was made to determine the probability of event occurring for a predictor and then statistically significant predictors were included in a multiple logistic regression analysis to evaluate the independent predictor value for each factor. A multivariable Cox regression analysis was performed to identify predictors of in-hospital all-cause mortality (only the first 120 days of survival were taking into considering). A *p* < 0.05 was considered statistically significant.

## 3. Results

From 116 patients initially selected, only 92 patients with definite infective endocarditis (IE) according to modified Duke criteria were included. Patients who didn’t fulfilled the criteria of definite endocarditis according to modified Duke criteria were excluded.

### 3.1. Patient Demographic Data and Characteristics

**Figure 1:**
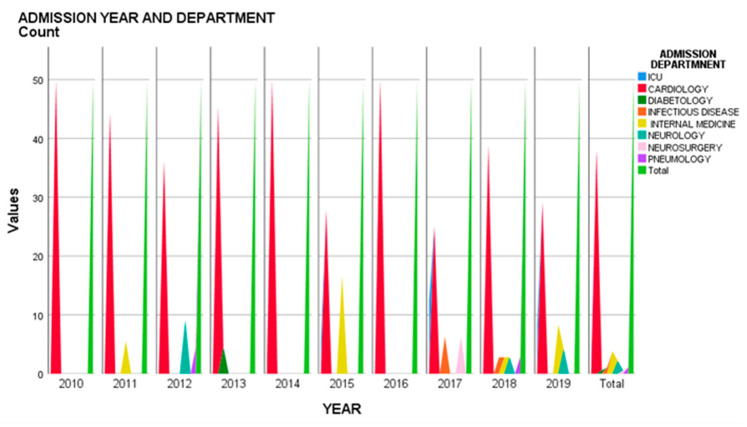
Admission year and department.

The mean age of patients was 63.80 ± 13.45 years, half of them being older than 65 years. Forty-nine patients were male (53.3%). Clinical symptoms were very variable so diagnosis was difficult. According to that a number of 70 patients (76.08%) were admitted to cardiology and 5 patients (5.43%) to the intensive care unit (ICU). The rest of 18 patients (19.56%) were initially admitted to other departments, seven (7.60%) of them to internal medicine, four (4.43%) of them to the neurology department, two (2.17%) of them to the infectious disease department, two (2.17%) of them to pneumology, one (1.08%) to the diabetology department and one (1.08%) to the neurosurgery department. There was a mean number of 9.2 ± 4.51 patients admitted/year with definite IE, the fewest were in 2010 and the most in 2018 (Figure 1).

### 3.2. Clinical Characteristics

The most common clinical symptoms and signs were: shortness of breath (75 patients, 81.5%), cardiac murmur (77 patients, 83.7%), fever (61 patients, 66.3%). Other important clinical signs were: pallor (49 patients, 53.3%), weight loss (39 patients, 42.4%) and anorexia (28 patients, 30.4%). Cerebral embolic events at admission were present in 4 patients (4.3%) admitted to the neurology department with ischemic embolic stroke. Sixty-six patients (71.7%) had native valve endocarditis, 26 patients (27.2%) had prosthetic valve endocarditis. One patient in the prosthetic valve group had congenital heart disease, surgically treated for Fallot tetralogy in childhood with closure of the ventricular septal defect with a prosthetic patch and reconstruction of the right ventricular outflow tract. He developed endocarditis at the level of a residual ventricular septal defect. The most frequently affected native valve was the mitral valve followed by aortic valve. Four patients (4.4%) had multiple valve involvement (Table 1).

The most frequently involved prosthetic valve was aortic mechanic valve followed by mitral mechanic valve. Multiple prosthetic valve involvement was present in 4 patients (4.4%) as can be seen in the Table 2. A VVI (Ventricular demand pacing) pacemaker was present in two patients with endocarditis: one with vegetations on the mitral valve and the other with biologic aortic and mitral prosthetic valve involvement. Device related endocarditis could not be confirmed.

### 3.3. Biological Features

**Figure 2:**
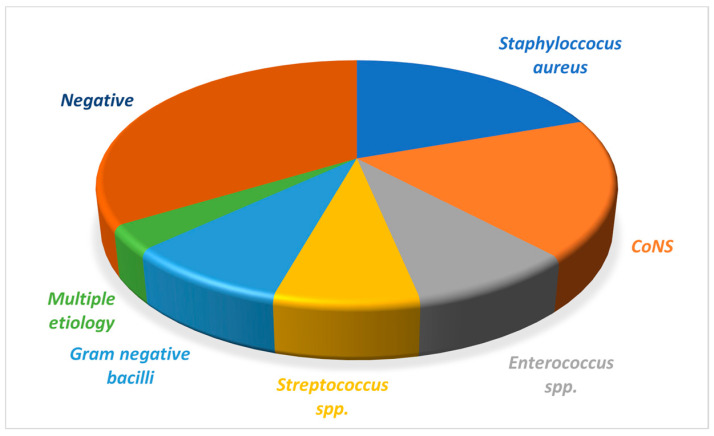
Infective endocarditis etiology.

Blood cultures were negative in 28 patients (30.43%) and positive in 64 patients (69.56%). The gram-positive aetiology was predominant (58.69%). Among Gram positive culture, staphylococcus group was the most frequently encountered (41 strains), 20 patients had positive blood cultures for *Staphylococcus aureus* (21.73%) and 21 patients had coagulase negative *Staphylococcus* (22.82%). *Enterococcus* spp. were isolated in 7 patients (7.6%) same as gram-negative bacillus (one with *Haemophilus influenzae* and six with non-HACEK group). Non-HACEK Gram-negative bacillus endocarditis were *Escherichia coli* (3 patients), *Klebsiella pneumoniae* (2 patients) and *Pseudomonas aeruginosa* (1 patient). All these patients had native valves IE and vascular catheterization in previous week. Seven patients had positive cultures for *Streptococcus* spp. (7.6%). They are all part of the viridans group (five strains were *Streptococcus gallolyticus*, two patients with *Streptococcus viridans*). In 3 patients, multiple germs (polymicrobial growth) were identified from blood cultures (3.3%) (Figure 2).

### 3.4. Health Care Associated Endocarditis

There were a number of 30 patients (32.6%) with health care associated invasive procedure performed in the six months before diagnosis of endocarditis: 24 patients (80%) had procedure performed during hospitalization-nosocomial endocarditis and 6 patients (20%) had out of hospital interventions -non nosocomial health care associated endocarditis. In the majority of cases (66.66%) vascular portal of entry was suspected (Table 3). There were no intravenous drug abusers in our cohort.

### 3.5. Imaging Evaluation

Electrocardiography was abnormal in 42 patients (45.7%), 28 patients (30.4%) with native valve endocarditis and 14 patients (15.2%) with prosthetic valve endocarditis (*p* = 0.325 between groups).

Most frequently encountered electrocardiographic findings were: left ventricular hypertrophy in 20 patients (21.7%), permanent atrial fibrillation in 20 patients (21.7%), paroxysmal atrial fibrillation in 9 patients (9.8%), intraventricular conduction abnormalities in 8 patients (8.6%) and atrioventricular conduction abnormalities in 6 patients (6.5%).

Left ventricular hypertrophy was more common in native valve endocarditis, 18 patients (19.6%) versus only 2 patients (2.2%) with prosthetic valve endocarditis (*p* = 0.041).

Paroxysmal atrial fibrillation was registered in 7 patients (7.6%) with native valve endocarditis versus 2 patients (2.2%) with prosthetic valve endocarditis (*p* = 0.67). Permanent atrial fibrillation, was more common in patients with prosthetic valve endocarditis-11 patients (12%) vs. 9 patients (9.8%) with native valve endocarditis (*p* = 0.003).

Atrioventricular block was more common in patients with prosthetic valve endocarditis, 4 patients (4.3%) versus only 2 patients (2.2%) with native valve endocarditis (*p* = 0.032).

Right bundle branch block was observed in 4 patients (4.3%), 3 patients (3.3%) with native valve endocarditis and one patient (1.1%) with prosthetic valve endocarditis (*p* = 0.884); left bundle branch block was registered in 4 patients (4.3%), 2 patients with native valve endocarditis (2.2%) and 2 patients (2.2%) with prosthetic valve endocarditis (*p* = 0.329).

Sustained ventricular tachycardia occurred in 2 patients (2.2%), one patient (1.1%) in the native valve endocarditis group and the other in the prosthetic valve endocarditis group (*p* = 0.45).

Transthoracic echocardiography was performed in all patients and transesophageal echocardiography was performed in 35 patients (38%). Vegetations as major criterion for diagnosis were identified in 82 patients (89.1%). Large vegetations >10 mm were present in 39 patients (42.4%) and intracardiac abscess was found in14 patients (15.2%). Twenty-six patients (28.6%) developed acute valvular regurgitation and seven patients (7.6%) presented prosthetic valve dysfunction.

### 3.6. Complications

In hospital follow-up complications were registered in 64 patients (69.6%). Heart failure was the most frequently encountered complication in 48.9% of patients. Eighteen patients (19.6%) had cardiogenic shock or acute pulmonary oedema, and 29.3% were in congestive heart failure. Factors that correlated with congestive heart failure by bivariate analysis were Charlson’s index (r = 0.291, *p* = 0.005) and *Staphylococcus aureus* etiology (r = 0.224, *p* = 0.032). In multivariate logistic regression independent predictors of congestive heart failure remained Charlson’s index (*p* = 0.007, OR = 1.435, 1.104–1.865) and *Staphylococcus aureus* infection (*p* = 0.035, OR = 3.324, 95% CI = 1.086–10.175).

Cardiogenic shock/acute pulmonary edema correlated in bivariate analysis with acute valvular regurgitation (r = 0.358, *p* = 0.001), acute renal failure (r = 0.344, *p* = 0.001) and septic shock (r = 0.429, *p* < 0.001). Independent predictors of cardiogenic shock/acute pulmonary oedema after multivariate logistic regression analysis remained: acute valvular regurgitation (*p* = 0.003, OR = 5.919, 95% CI = 1.797–19.493) and acute renal failure (*p* = 0.003, OR = 5.919, 95% CI = 1.797–9.493).

Acute renal failure occurred in 27 patients (29.3%) and correlated in bivariate analysis with septic shock (r = 0.355, *p* = 0.001) and cardiogenic shock (r = 0.344, *p* = 0.001). In multiple logistic regression these factors remained independent predictors: septic shock (*p* = 0.033, OR = 4.58, 95% CI = 1.133–18.520) and cardiogenic shock (*p* = 0.038, OR = 3.572, 95% CI 1.073–11.893).

Embolic events were registered in 19 patients (20.7%) (12 cerebral, 3 spleen, 1 peripheral artery, 3 coronary arteries). All three patients with coronary artery embolism presented with ST elevation myocardial infarction and had native valve endocarditis, two patients with aortic valve involvement and one patient with mitral valve involvement. The coronary arteries affected were middle left anterior descending artery in two patients and posterolateral branch of the circumflex artery in one patient. Blood cultures were positive for Staphylococcus aureus in one patient, CoNS (Coagulase negative *Staphylococcus*) in one patient and were negative in one patient. These events correlated with large vegetations (r = 0.323, *p* = 0.002) by bivariate analysis. In multivariate logistic regression analysis only vegetation size remained independent predictor of embolic events (*p* = 0.009, OR = 4.659, 95% CI = 1.472–14.746).

Twelve percent of patients had ischemic stroke and one patient developed cerebral hemorrhage. Ischemic stroke was correlated with health care associated IE (r = 0.350, *p* = 0.001), *Streptococcus gallolyticus* IE (r = 0.474, *p* < 0.001) and in general *Streptococcus* spp. etiology (r = 0.254, *p* = 0.015). In multivariate logistic regression analysis *Streptococcus* spp. etiology remained an independent predictor of ischemic stroke (*p* = 0.028, OR = 6.333, 95% CI 1.218–32.935).

Other complications were intracardiac abscess in 15.2% of patients and septic shock in 14.1% of patients. Prosthetic valve dysfunction occurred in 7.6% of patients. Intracardiac abscess correlated directly with the presence of prosthetic valve (r = 0.272, *p* = 0.009) and development of acute valvular regurgitation (r = 228, *p* = 0.029). In multivariate logistic regression analysis, the most important predictor of intracardiac abscess remained *Staphylococcus aureus* infection (*p* = 0.002, OR = 10.334, 95% CI 2.425–44.036). Septic shock correlated with gram negative bacilli etiology (r = 0.207, *p* = 0.048) and acute renal failure (r = 0.355, *p* = 0.001) in bivariate analysis. In multivariate logistic regression analysis independent predictor for septic shock was acute renal failure (*p* = 0.002, OR = 7.853, 95% CI 2.078–29.681).

Atrioventricular conduction abnormality appeared in 6.5% of patients and correlated with the presence of a prosthetic valve (r = 0.225, *p* = 0.031), acute renal failure (r = 0.216, *p* = 0.038), intracardiac abscess (r = 0.256, *p* = 0.014), and prosthetic valve dysfunction (r = 0.256, *p* = 0.014) in bivariate analysis. Complete atrioventricular block occurred in 3.3% of patients, second degree atrioventricular block 2:1 was present in 1.1% of patients and 2.2% of patients had first degree atrioventricular block. In multivariate regression analysis intracardiac abscess was independent predictor for atrioventricular conduction abnormality (*p* = 0.048, OR = 6.148, 95% CI 1.017–37.147).

Mean age of patients with surgical treatment was 64.03 ± 14.49 years. From 32 patients who underwent surgery a number of six had prosthetic valve endocarditis. Most of them, 28 patients (30.4%) had valve replacement vs. only three patients (3.3%) with valve repair (Table 4). One patient with severe aortic stenosis and associated high surgical risk underwent transaortic valve implantation after healing of endocarditis (1.1%). Mean hospital stay in surgery was 14.3 ± 11.7 days.

Surgery was recommended in 62 patients (67.4%), more often in IE with *Staphylococcus aureus* etiology (r = 0.285, *p* = 0.006); *Staphylococcus* spp. infection (r = 0.259, *p* = 0.013) in patients with large vegetations (r = 0.550, *p* < 0.001) and in acute valvular regurgitation (r = 0.392, *p* < 0.001). It was performed on an emergency basis due to hemodynamic instability in 8 patients (8.7%) and was elective in 24 patients (26%). The rest of 30 patients did not undergo surgical intervention due to various reasons: patient refusal, surgeon deny, poor clinical condition or were lost from observation. Mortality after surgery was 18.75% (6/32), 37.5% (3/8) for patients operated in emergency versus 12.5% (3/24) for patients with elective surgery (*p* = 0.3). Causes of mortality for patients operated in emergency were: systemic sepsis and multiple organ failure for two patients and bleeding with cardiac tamponade in one patient. For patients with elective surgery the causes of death were: cardiogenic shock in two patients and hemorrhagic stroke in one patient. Mortality was significantly higher in the group with prosthetic valve endocarditis who underwent surgery 50% (3/6) vs. 11.54% (3/26) in the native valve endocarditis group (*p* = 0.03). Postoperative complications appeared in 53.13% (17/32) of patients. Major postoperative complications are presented in Table 5.

Surgery in emergency correlated with acute valvular regurgitation (r = 0.233, *p* = 0.026), elective surgery correlated directly with the presence of large vegetations (r = 0.245, *p* = 0.018) and inversely with stroke (r = −0.256, *p* = 0.014) and septic shock (r = −0.268, *p* = 0.01). An independent predictor for elective surgery in multivariate regression was the presence of large vegetation (*p* = 0.023, OR = 3.062 95% CI 1.170–8.014)

### 3.7. Mortality

**Figure 3:**
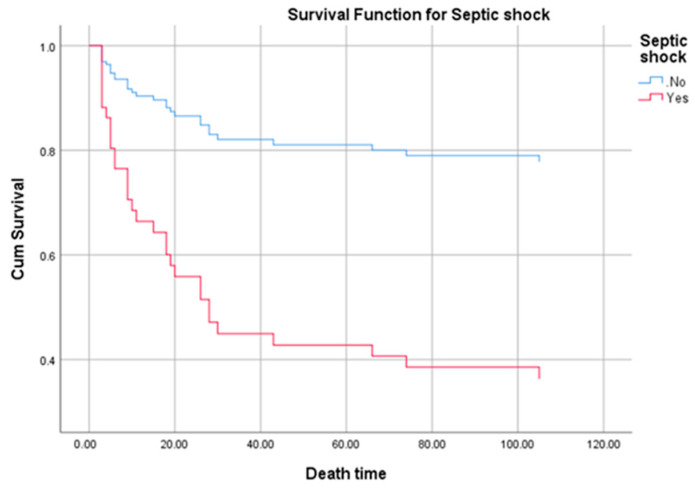
Survival curve of patients with septic shock for the first 120 days shows a significant lower survival compared to patients without septic shock (*p* < 0.001).

Death occurred in 31 of patients which accounts for a mortality of 33.7%. Death correlated in univariate Cox regression analysis with: *Staphylococcus aureus* etiology (*p* = 0.04, OR = 2.207, 95% CI 1.036–4.700), prosthetic valve dysfunction (*p* = 0.024, OR = 3.04, 95% CI 1.161–7.96), embolic events (*p* = 0.02, OR = 2.403, 95% CI 1.148–5.028), cardiogenic shock (*p* = 0.001, OR = 3.518, 95% CI 1.702–7.275), congestive heart failure (*p* = 0.018, OR = 2.365, 95% CI 1.162–4.812), acute renal failure (*p* < 0.001, OR = 4.181, 95% CI 2.040–8.568), stroke (*p* = 0.004, OR = 3.147, 95% CI 1.441–6.872) and septic shock (*p* < 0.001, OR = 8.091, 95% CI 3.861–16.953).The overall survival of patients with septic shock can be seen in the Figure 3.

**Figure 4:**
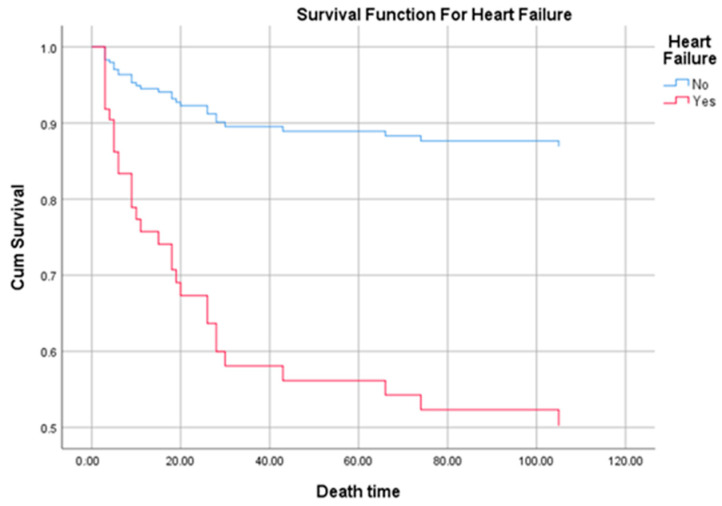
Survival curve of patients with heart failure.

Independent predictors for death in multivariate cox regression analysis remained: congestive heart failure (*p* = 0.025, OR = 2.270, 95% CI 1.108–4.651) and septic shock (*p* < 0.001, OR = 7.950, 95% CI 3.769–16.767). Survival of patients with heart failure for the first 120 days shows a significant lower survival compared to patients without heart failure (*p* = 0.02) as can be seen in the Figure 4.

### 3.8. Etiologic Correlations with Different Parameters

#### 3.8.1. Etiologic Correlations with Demographic Data

There were no significant differences between etiology groups regarding age or sex. Charlson’s comorbidity index was greater in the *Enterococcus* group, where patients were also older and although there was a significant correlation between Charlson’s index and enterococcal infection (r = 0.235, *p* = 0.024) there was no statistically significant difference between groups. A statistically significant difference was observed regarding antibiotic therapy prior to admission between different etiologic groups, respectively in the negative blood culture group significantly more patients took antibiotics before blood cultures were drawn (Table 6).

#### 3.8.2. Etiologic Correlation with Clinical Data

There was a significant direct bivariate correlation between *Staphylococcus aureus* infection and fever (r = 0.294, *p* = 0.005) and an inverse correlation between negative blood culture and fever (r = −0.465, *p* < 0.001). At intergroup comparison fever was significantly more frequent in the *Staphylococcus* group. There were no other statistically significant differences regarding clinical symptoms and signs between groups. Prosthetic valve endocarditis correlated with *Staphylococcus* etiology (r = 0.353, *p* < 0.001). There was a statistically significant difference at intergroup comparison: patients with prosthetic valve IE were significantly more in the *Staphylococcus aureus* group. (Table 7)

#### 3.8.3. Etiologic Correlation with Imagistic Data

*Staphylococcus aureus* IE was associated with the presence of large (more than 10 mm) vegetations (r = 0.353, *p* = 0.001). There was a significant difference between etiologic groups regarding the presence of large vegetations, more patients in the *Staphylococcus aureus* group presenting with large vegetations. The presence of intracardiac abscess has a bivariate correlation with *Staphylococcus aureus* infection (r = 0.478, *p* < 0.001) and significantly more patients developed intracardiac abscess in the *Staphylococcus aureus* group. Prosthetic valve dysfunction occurred at significant different levels in different etiological groups, being more frequent in CoNS group (Table 8). There was a significant bivariate correlation between coagulase negative *Staphylococcus* infection and prosthetic valve dysfunction (r = 0.286, *p* = 0.006).

## 4. Discussion

Half of patients in our study were older than 65 years. Older patients have an increased incidence of bacterial endocarditis of about 9 time higher than lower groups of age [7,8]. Age distribution of patients with endocarditis has been changing lately. While in the past IE affected young adults with predominantly rheumatic valvular disease, it has become at present a disease that affects older patients often after therapeutic procedures. Patients are older since the survival of patients with rheumatic and congenital heart disease has improved and degenerative valvular disease is frequent in old people [8]. This finding is in agreement with results observed in other studies. EURO-ENDO registry confirms the increasing age of patients with IE and the progressively increasing age was observed also in three surveys conducted in French regions in 1991, 1999 and 2008 [2,9,10].

The distribution of patients regarding to sex in our study is approximatively 1/1, different from that described in several epidemiological studies where male sex appears to be more frequently involved [8]. The male/female ratio can exceed 2:1 with a range of 1 to 3:1 in different studies. Although the incidence is increased in male, prognosis appear to be worse in women [11,12].

Almost 84% of patients had associated comorbidities in our study, Charlson’s comorbidity index number was 3.53 ± 2.029. In general patients with IE are in present days older, frailer and with several comorbidities. Health care associated IE is increasing and new at-risk groups have appeared: patients with prosthetic valves and intracardiac devices [13]. Due to an increased number of invasive diagnostic and therapeutic procedures health care associated IE has a reported incidence from 7% to 34% according to various authors [14,15,16]. Health care associated IE was present in one third of patients in our study similar with reports from other authors. The epidemiological profile of IE has changed in the past years and it is now health care-acquired in more than 25% of cases according to Cahill [17]. In EURO-ENDO registry health care associated IE was present in a similar proportion, 32.96% of patients, nosocomial in 60.8% of them and no nosocomial in 39.2% of them [9]. Nosocomial IE was present in 80% of patients with health care associated IE in our study.

The source of infection was vascular in the majority (66.66%) of patients with health care associated IE similar results were found by by Lomas et al. where vascular source of infection was present in 63% of health care associated IE [14]. IE is heterogenous in clinical manifestation so almost 20% of patients were admitted initially to other departments. The EURO endo registry observed similar results: 8.8% of patients were recruited from the internal medicine ward and 9.3% from the infectious diseases ward [9]. A percentage of 76% of patients were admitted directly to cardiology in our study and approx. 68% of patients in EURO ENDO registry were recruited from cardiology related departments [9]. A number of 5.43% patients were critically ill and were admitted to the intensive care unit (ICU) significantly less than patients in EURO ENDO where 15% were admitted directly to the ICU [9].

The incidence of prosthetic valve endocarditis is increasing and was found also with increased frequency in 27.2% of cases in our study. EURO ENDO registry and Euro Heart Survey reported a similar proportion of prosthetic valve endocarditis as in our study (30% and respectively 26%) [9,18]. Older studies as the French registry in 2008 and the International Collaboration on Endocarditis-Prospective Cohort (ICEP) Study in 2009 reported a lower prevalence of prosthetic valve endocarditis of 25% and respectively 21% [10,19].

A very small number of patients with IE had devices in the present study. A VVI pacemaker was present in two patients with endocarditis both with vegetations on cardiac valves so device related endocarditis could not be confirmed. In EURO ENDO significantly more patients were diagnosed with device related endocarditis (9.9%), compared to ICEP study (7%) [9,19].

Aortic and mitral native valve were almost equally affected in our study. In 35.86% of patients the mitral valve was involved and in 31.52% the aortic valve was involved. Multiple valve involvement was present in in 4.4% of patients. The results are comparable to those observed in EUROENDO registry [9]. In a study performed in another region of Romania IE was localized on native aortic valve in 50%, mitral native valve in 40% and on prosthetic valve in 10% [20]. Mitral valve was significantly more frequently involved in the study performed by Khan et al. at Karachi National Institute but in this study, patients were significantly younger with increased frequency of rheumatic valve disease and mitral valve is most commonly affected in rheumatic valve disease [13].

Most common clinical symptoms and signs were: shortness of breath, cardiac murmur and fever. In EURO ENDO registry the most common clinical presentation consisted of fever (77.7%) and cardiac murmur (64.5%) [9]. More patients presented with fever and cardiac murmurs and less with dyspnea in the study performed by Khan et al. on 75 patients with IE, but patients in this study were significantly younger [13].

Cerebrovascular accident was the clinical presentation in 6.8% of patients in EURO ENDO registry [9]. A lower percentage of patients 4.35% presented with stroke on admission and were managed initially in the neurology department in the present study.

Blood cultures were negative in one third of the patients. A higher proportion of patients (79%) compared to our study had positive blood culture test in EURO ENDO registry and in European heart survey (86%) [9,18]. As in our study, several Indian authors reports low percentages for blood culture positivity ranging from 41% to 67% [21,22]. Ghosh et al. reported 52% positive blood cultures relying on standard culture techniques [23]. The results are consistent with those observed in other studies. The most frequent identified microorganism was also *Staphylococcus* spp. in EURO ENDO registry (44.1%). *Streptococcus* spp. and *Enterococcus* spp. were also less common identified, but with a higher frequency than in our study 19% and respectively 15.8% [9].

*Staphylococcus aureus* was the predominant microorganism identified in our study. The incidence of *Staphylococcus aureus* IE has increased and in the developed world has become the most common causative germ [17,24]. Ghosh et al. in a study performed in 2014 in India reported *Staphylococcus* spp. as the majority etiology although rheumatic heart disease was the major cardiac substrate at risk in this study [23]. Several studies performed in India revealed some temporal trends of IE etiology. While Garg et al. found a higher prevalence of *Streptococcus* IE (23%), Math et al. found equal incidence of *Staphylococcus* and *Streptococcus* etiology [21,22]. Other studies also revealed *Staphylococcus aureus* as the most frequent etiologic factor [25,26,27]. *Staphylococcus aureus* was found with increased frequency in patients with prosthetic valve endocarditis in our study. This microorganism has a predilection to adhere to prosthetic materials. Many other studies reported *Staphylococcus aureus* as the leading etiology of prosthetic valve endocarditis [28,29]. Barraua et al. reported as the commonest organism found in prosthetic valve endocarditis *Streptococcus* strains, especially *Streptococcus bovis*, while *Staphylococcus* spp. were second in frequency [30].

CoNS have become commonly isolated pathogens causing more than 10% of all IE cases [19,31,32]. CoNS account for approximately 25 to 48% of all cases of prosthetic valve endocarditis in various studies [30,33,34,35]. An increased frequency of CoNS infection was present in our study; it affects 26.92% of patients with prosthetic valve IE and is frequently associated with prosthetic valve dysfunction.

Negative blood culture is now less prevalent since the advent of serological and molecular diagnostic techniques which allow identification of a lot of fastidious microorganisms [24]. Blood culture-negative endocarditis may account for 2.5% to 70% of all cases of endocarditis, with geographical variation in incidence that may be explained by several factors including: specific epidemiological factors, as is the case for fastidious zoonotic agents; variations in use of antibiotics prior to blood drawing; differences in testing strategies and involvement of unknown pathogens [36]. In the present study negative blood culture test was significantly associated with antibiotic administration prior to admission. Identification of pathogen is essential in the diagnosis and treatment of IE but failing in identification of etiologic agent is related most frequently to previous antibiotic treatment. Other studies revealed also the consequences of antibiotic treatment prior to blood drawn for cultures. In the study performed by Lamas et al. from 63 cases of culture negative IE in half of them previous antibiotic treatment was administered and among 73 cases without etiology 58 received antibiotic prior to treatment in the study performed by Houpikinian et al. [37,38]. The rate of culture neg IE will decrease with systematic use of specific serology in all cases and with increasing use of newer histological and molecular tests on blood and tissue samples [36].

Echocardiography is the most used imaging method of diagnosis [39]. Transthoracic echocardiography was performed in all patients and transesophageal echocardiography was performed only in 35 patients (38%), 48.6% of them in patients with prosthetic valves. Significantly more patient underwent transesophageal echocardiography in EURO-ENDO registry (58.1%), predominantly in suspected prosthetic valve EI. Vegetations as major criterion for diagnosis was identified in the majority of patients with large vegetations and intracardiac abscess. In EURO ENDO registry vegetation were found in 72.7% of patients and cardiac abscess was present in 17.8% of patients, similar percentages as in our study [9]. *Staphylococcus aureus* etiology was found to be associated with large vegetations and intracardiac abscess in our study. These results were also observed in other investigations [40].

The most frequent complications observed in our study were: heart failure and acute renal failure. Congestive heart failure was reported in one third of patients and cardiogenic shock and/or acute pulmonary oedema was registered in another 19.6% of patients. EURO ENDO registry observed a lower percentage of congestive heart failure (14.1%) and of cardiogenic shock (6.7%). In our study the most important predictors of congestive heart failure were Charlson’s index and *Staphylococcus aureus* etiology. Acute renal failure was present in 29.3% of patients enrolled in the present study significantly more than patients from EURO ENDO study (17.7%) [9]. In EURO ENDO registry the most frequent complication were embolic events observed in up to 40% of patients. From them 44. 1% were with cerebral localization. A lower rate of embolic events was observed in our study 2.7%, similar with percentage reported in ICE (International Collaboration on Endocarditis) cohort 23% and 18% in Euro Heart Survey [18,19]. Septic shock occurred in 14.1% of patients, higher percentage than that observed in EURO ENDO 9.5% [9]. Gram negative bacilli etiology IE correlated with septic shock in our patients and this result was also observed in the study performed by Veve et al. where 44% of patients with this etiology were in septic shock [41]. AV (Atrio-ventricular) block was observed in 4.5% in EURO ENDO and a similar value was reported in the present study 6.5% and it was correlated with CoNS IE etiology.

Despite advances in diagnosis and treatment, incidence and mortality of the disease did not decrease, and mortality had reached 20% in the last 30 years [8]. A high percentage of death was observed in our study in 33.7% of patients. Some authors also report high rates of mortality between 15 to 30% [1]. Death occurred only in 17% of patients in EUROENDO registry [9]. In ICE cohort mortality was 18% [20] and in Euro heart survey was 12.7% [19] significantly less than that observed in our study. However, the number of patients was lower in our study and only patients with definite diagnosis of IE were included. Independent predictors of mortality in EURO ENDO were heart failure, cerebral complications, abscess, large vegetations, Charlson’s index and failure to undertake surgery despite guidelines recommendation [9]. In the present study congestive heart failure and cardiogenic shock showed independent prediction of death.

*Staphylococcus aureus* infection was correlated with death in univariate cox regression analysis in our study and also in EURO ENDO registry [9]. *Staphylococcus aureus* patients have more severe IE compared to non-*Staphylococcus aureus* patients, with higher rates of preoperative vascular complications, septic shock, embolic events, stroke, and annular abscess [42]. *Staphylococcus aureus* IE is associated with more severe prognosis than other pathogens (excluding fungal pathogens), with a high in-hospital mortality of 30% to 46%, according to various authors [10,42,43].

Surgery was recommended in 67.4% of patients but was performed only in 36.95% of them. Following ESC guidelines surgery was recommended in a similar percentage 69.3% of patients in EURO ENDO registry but was performed in significantly more patients 51.2% than in our study [8]. Cardiovascular surgery was performed on an emergency basis due to hemodynamics instability in 8 patients in general patients with severe heart failure due to acute valvular regurgitation (8.7%) and was elective in 24 patients (26%). The rest of the 30 patients did not undergo surgical intervention due to various reasons: patient refusal, surgeon deny, poor clinical condition or were lost from observation. Patients with severe medical condition with stroke or septic shock were less surgically managed. Elective surgery was more frequently performed in patients with large vegetations and with *Staphylococcus aureus* etiology. In EURO ENDO registry in general factors associated with cardiac surgery were congestive heart failure, vegetation length, abscess and cerebral complications [9].

## 5. Conclusions

Endocarditis affected predominantly older age. Prosthetic valve endocarditis and health care associated endocarditis are increasing. We observed an increased frequency of staphylococcal IE, caused by both *Staphylococcus aureus* and CoNS and a low incidence of streptococcal and enterococcal etiology. Negative blood culture is still frequently encountered.

*Staphylococcus aureus* is more frequent in prosthetic valves IE and is associated with large vegetations and intracardiac abscess. *Staphylococcus aureus* infection is severe with complications as congestive heart failure and embolic events. Moreover, death appears correlated with *Staphylococcus aureus* etiology. Prosthetic valve dysfunction is frequently associated with CoNS etiology. *Streptococcus gallolyticus* although rarely encountered was complicated often with large vegetations and stroke. *Enterococcus* infection appeared more frequently in patients with high comorbidity index. An increased incidence of gram-negative bacillus IE was found with severe clinical evolution complicated often with septic shock.

## Figures and Tables

**Table 1:** Endocarditis localization native valve.

Valves	Number (Percent)
Native aortic valve	29 (31.5)
Native aortic and tricuspid valve	2 (2.2)
Native mitral valve	33 (35.9)
Native mitral and aortic valve	2 (2.2)
Total	66 (71.7)

**Table 2:** Endocarditis localization prosthetic valve.

Valves	Number (Percent)
Prosthetic aortic biologic valve	3 (3.33)
Prosthetic aortic mechanic valve	10 (10.9)
Prosthetic mitral biologic valve	1 (1.1)
Prosthetic mitral mechanic valve	7 (7.6)
Prosthetic mitro-aortic biologic valve	3 (3.33)
Prosthetic mitro-aortic mechanic valve	1 (1.1)
Corrected Tetralogy Fallot	1 (1.1)
Total	26 (27.2)

**Table 3:** Health care associated procedures.

Medical Procedure	*n* = 30	(%)
Vascular peripheral vein catheter	9	9.78
Vascular central vein catheter	1	1.08
Vascular hemodialysis	2	2.17
Vascular cardiac surgery	3	3.26
Vascular angiography	3	3.26
Vascular surgery	2	2.17
Gastrointestinal	3	3.26
Brain surgery	2	2.17
Dental	2	2.17
Skin	1	1.08

**Table 4:** Type of surgery performed.

Operative Procedure	Patients (No/%)	Type of Implanted Valves
Aortic valve replacement	17 (18.5%)	
	12 (13%)	mechanical
	5 (5.4%)	bioprosthetic
Mitral valve replacement	9 (9.8%)	
	5(5.54%)	mechanical
	4 (4.3%)	bioprosthetic
Double valve replacement (mitral and aortic)	2 (2.2%)	
	1 (1.1%)	mechanical
	1 (1.1%)	bioprosthetic
Mitral valve repair	3 (3.3%)	

**Table 5:** Major complications after surgery.

Complication	Number of Patients
Cardiogenic shock	5 (15.62%)
Bleeding requiring reintervention	6 (18.75%)
Stroke	2 (6.25%)
Atrioventricular block	2 (6.25%)
Renal failure	7 (21.88%)
Mediastinitis	4 (12.8%)
Systemic sepsis	3 (9.38%)

**Table 6:** Etiologic correlations with demographic data.

Demographic Data	Age	Sex (M) Number (%)	Charlson Index	Health Care Associated IE Number (%)	Antibiotherapy Prior Blood Culture Number (%)	Admission to Other Departments Number (%)
TOTAL (*n* = 92)	63.80 ± 13.45	49 (53.3)	3.53 + 2.02	30 (32.6)	25 (27.2)	22 (23.9)
*Staphyloccocus aureus* (18)	61.16 + 14.15	10 (55.6)	3.66 + 1.71	8 (44.4)	2 (11.1)	7 (38.9)
CoNS (17)	61.11 + 16.26	12 (70.6)	3.35 + 2.66	6 (35.3)	3 (17.6)	3 (17.6)
*Enterococcus* spp. (8)	70.87 + 8.23	3 (37.5)	5 + 1.41	5 (62.5)	1 (12.5)	2 (25)
*Streptococcus* spp. (7)	60.14 + 13.69	6 (85.7)	2.57 + 1.39	2 (28.6)	2 (22.6)	2 (28.6)
Gram negative bacilli (8)	64.62 + 14.22	1 (12.5)	3.62 + 1.99	3 (37.5)	1 (12.5)	0
Multiple etiology (3)	67.66 + 7.76	1 (33.3)	3.33 + 2.51	0	0	0
Negative (31)	65.22 + 12.69	16 (51.6)	3.38 + 1.99	6 (19.4)	16 (51.6)	8 (25.8)
*p*	0.591	0.066	0.412	0.207	0.014	0.41

CoNS: Coagulase negative *Staphylococcus*.

**Table 7:** Etiologic correlation with clinical data.

Clinical data	Fever	Dyspnea	Murmur	Anorexia	Weight loss	Pallor	Prosthetic Valve
	Number (%)						
TOTAL (*n* = 92)	61 (66.3)	75 (81.5)	77 (83.7)	28 (30.4)	39 (42.4)	49 (53.3)	26 (28.3)
*S. aureus* (18)	17 (94.4)	16 (88.9)	14 (77.8)	6 (33.3)	10 (55.6)	10 (55.6)	10 (55.6)
CoNS (17)	14 (82.4)	13 (76.5)	14 (82.4)	6 (45.3)	8 (47.1)	9 (52.9)	7 (41.2)
*Enterococcus* spp. (8)	7 (87.5)	7 (87.5)	8 (100)	2 (25)	2 (25)	4 (50)	1 (12.5)
*Streptococcus* spp. (7)	4 (57.1)	5 (71.4)	6 (85.7)	3 (42.9)	4 (57.1)	5 (7.4)	0
GN bacilli (8)	6 (75)	8 (100)	7 (87.5)	2 (25)	2 (25)	3 (37.5)	0
Multiple etiology (3)	2 (66.7)	1 (33.3)	3 (100)	2 (66.7)	3 (100)	2 (66.7)	1 (33.3)
Negative (31)	11 (35.5)	25 (80.6)	25 (80.6)	7 (22.6)	10 (32.3)	16 (51.6)	7(22.6)
*p*	<0.01	0.241	0.830	0.727	0.153	0.923	0.014

**Table 8:** Etiologic correlation with imagistic data.

Echocardiography	Vegetation	Large Vegetation	Acute Valvular Regurgitation	Intracardiac Abscess	Prosthetic Valve Dysfunction
Number (%)					
TOTAL (*n* = 92)	82 (89.1)	39 (42.4)	26 (28.6)	14 (15.2)	7 (7.6)
*Staphylococcus aureus* (18)	13 (72.2)	14 (77.8)	6 (35.3)	9 (50)	3 (16.7)
CoNS (17)	15 (88.2)	6 (35.3)	4 (23.5)	3 (17.6)	4 (23.5)
*Enterococcus* spp. (8)	8 (100)	3 (37.5)	4 (50)	0	0
*Streptococcus* spp. (7)	7 (100)	4 (57.1)	4 (57.1)	0	0
Gram negative bacilli (8)	7 (87.5)	3 (37.5)	1 (12.5)	0	0
Multiple etiology (3)	3 (100)	0	0	0	0
Negative (31)	29 (93.5)	9 (29)	7 (22.6)	2 (6.5)	0
*p*	0.232	0.018	0.249	<0.001	0.04
